# Online exercise program for men living with obesity: Experiences, barriers, and enablers

**DOI:** 10.1016/j.conctc.2023.101226

**Published:** 2023-11-10

**Authors:** Lisa Thomson, Mohammad Keshavarz, Martin Sénéchal, Danielle R. Bouchard

**Affiliations:** a University of New Brunswick, Department of Sociology Fredericton NB, Canada; b University of New Brunswick, Faculty of Kinesiology Fredericton NB, Canada; c Cardiometabolic Exercise and Lifestyles Laboratory, Fredericton NB, Canada

**Keywords:** Male, Physical activity, Web, Compliance

## Abstract

The prevalence of obesity is increasing among men, and this population remains under-represented in lifestyle and weight management interventions. The current study aims to explore the experiences of men living with obesity (body fat ≥25 %) toward a 12-week supervised online exercise platform. Ten men were interviewed for this qualitative study. Semi-structured, open-ended phone interviews were conducted, and the transcripts were thematically coded using the qualitative data analysis Nvivo QSR software package. The research findings are illustrated using quotes from participants. The results were organized into two main themes: those that removed barriers to exercise and those that improved the enablers of exercise. Eliminating barriers included not purchasing specialized equipment or travelling to a gym facility. The enablers to their success with the program included the structured format of the circuit program and having supervised sessions. By removing barriers and enhancing enablers, the 12-week online exercise circuit program increased compliance to and success of the exercise program for men living with obesity. Future research should explore the long-term effects of an online program for men living with obesity and its appeal beyond COVID-19.

## 1 Introduction

The prevalence of obesity quantified by body mass index, body fat % or waist circumference has increased more in males than females in the past decades [1]. For example in Europe, it is predicted that 60 % of males and 50 % of females will live with obesity by 2050 [1]. Similarly, sex differences in obesity prevalence were also reported in Canada (69.4 % of males vs. 56.7 % of females in 2018) [2].

Despite the well-documented benefits associated with exercise [3], only about 20 % of adults meet the minimum weekly recommendations, which is a minimum of 150 min of moderate-to-vigorous aerobic activities and at least two strength conditioning sessions [4]. This proportion is even lower for specific groups, such as those living with obesity [5]. Exercising with peers of similar age and gender is seen to be encouraging for adults living with obesity [[6], [7], [8], [9], [10], [11]], suggesting that feeling emotionally secure and accepted cannot be underestimated when developing exercise programs targeting people living with obesity [12]. It has become increasingly evident that creating an environment that addresses the barriers faced by individuals living with obesity when they aspire to engage in physical activity plays a key role in motivating them to participate in regular exercise [13]. The significance of this emotional component cannot be overstated, as it could serve as a cornerstone in the design and development of exercise programs tailored to meet the unique needs of individuals within this demographic [14].

The prevalence of obesity is high for both men and women and is rising particularly among men, along with more chronic health conditions [15]. However, men are usually under-represented in lifestyle and weight management interventions [16]. A systematic review of 237 studies [17], showed that only 27 % of men participate in weight management programs, and only 5 % of all studies recruited men. Evidence suggests that men's attitudes, experiences, expectations, and feelings toward the nature of a program can have a decisive impact on their participation [18]. It is emphasized that weight management programs in a positive environment with a certain masculine culture could be successful when targeting men living with obesity, such as programs in partnership with sports clubs while targeting men only [19,20]. Participating in a lifestyle program exclusively for men would be appealing, especially to those with similar interests and social and cultural backgrounds [[6], [7], [8], [9], [10], [11]].

COVID-19 forced people to engage in various online exercise programs that they may not have normally [21,22]. This online mode of delivery addresses some common barriers to exercise, such as transportation, accessibility, cost, and convenience, and specific to people living with obesity: the shame of their body (feeling self-conscious or embarrassed about their physical appearance in public places) and fitness levels and the perception that they are being judged by others (being scrutinized or evaluated by other people, possibly in a negative or judgmental way) [23]. A systematic review indicated that internet-based interventions significantly improved adult physical activity levels [24]. Despite the value of internet-based interventions, a study has reported that only about a third of participants in such programs are men [25]. By examining the potential benefits of an online exercise program for individuals living with obesity, specifically by addressing their barriers to regular exercise, this research aims to fill a crucial gap in the current literature. The study's findings could offer valuable insights into designing effective, gender-specific interventions that prioritize emotional well-being and cultivate a supportive exercise environment for men living with obesity, ultimately contributing to enhanced public health outcomes.

There is currently a lack of studies targeting males living with obesity and their personal experiences with an online exercise program [26]. In the current study, we offered a structured and supervised online 12-week circuit training program to men living with obesity. This qualitative study aimed to explore the barriers and enablers to exercise for men living with obesity who participated in an online exercise program. The objective of this study is exploratory, taking an inductive approach to understanding further men's experiences in a 12-week online exercise platform designed specifically for them.

## 2 Methods

Participants interviewed in this qualitative study were originally enrolled in a larger randomized control trial, registered at ClinicalTrials.gov (NCT04679090), which involved recruiting 60 men with obesity (body fat ≥25 %) for a 12-week intervention. Participants were eligible if they 1) characterized themselves as men; 2) 19 years or older, 3) living with obesity defined as a body fat % equal or above 25 kg/m^2^ [27] confirmed by a BodPod (COSMED, Rome, Italy) confirmed at the baseline visit, and 4) having access to home internet and an electronic device. The primary objective was to investigate the impact of an online circuit exercise program on adherence to weekly physical activity guidelines (≥150 min of moderate to vigorous aerobic exercise, plus at least two sessions of muscle-strengthening activities) 24 weeks after being exposed to the intervention compared to a control group [28]. The circuit consisted of four basic exercises (squats, push-ups, lunges and triceps dips) conducted three times a week for 50 min per session, where the goal was to do as many repetitions as possible in 45 s for each exercise. We found that the proposed program increased adherence to the physical activity guidelines after about a year for men living with obesity. As a secondary objective, we aimed to understand participants' experiences by conducting interviews at the end of the intervention (after 12 weeks). The interview guide was developed based on the transtheoretical model, which stipulates that behaviour change follows different steps based on the individual's readiness to act. All interviews were done in English by the same person (LT) using the standard steps of thematic analysis [28]: Familiarizing with the data, generating initial codes, searching for themes, reviewing themes with MK, and DRB, defining themes, and reporting the findings.

### 2.1 Participants

All participants in the intervention arm (n = 30) were offered to be in the qualitative study at the end of the intervention. A total of 10 men accepted to be interviewed. Based on the transtheoretical model, participants were in either the contemplation or the action phase with the goal of bringing them to the maintenance phase. Given the COVID-19 environment, semi-structured, open-ended phone interviews were conducted during the summer of 2021 with the male participants 12 weeks after starting the intervention. The interviews were based on audio-recorded and professionally transcribed verbatim.

The duration of the interviews was between 18 and 40 min, with an average length of 28 min. Participants ranged in age from 22 to 58, with a mean age of 41.2 (SD 11.0). Seven participants were employed full-time, two were retired military, and one was a full-time student. All participants were given pseudonyms chosen by the research team.

Given the limited research documenting men's experiences with obesity, a qualitative, exploratory research design was utilized. Our exploratory study provides insights into the experiences of men living with obesity while engaged in an online circuit-style exercise program. The quotes and data presented in our study aim to illustrate these participants' experiences and to demonstrate where similarities converge. The interviews were conducted over the phone due to COVID protocols in place at the time. Although written consent was obtained for the original intervention when participants came for assessment, the informed consent document for the qualitative objective was read, and audio recorded by each participant along with their response before the interview was conducted. Verbal informed consent was obtained before the interviews, and the Research Ethics Board approved all procedures at the University of New Brunswick (REB #2020-008).

### 2.2 Intervention

**Fig. 1:**
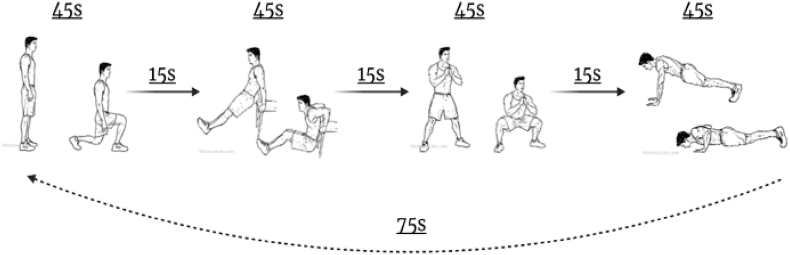
The four prescribed exercises.

Participants were asked to exercise three times a week and perform four basic resistance exercises in a circuit format for 12 weeks while supervised via the Microsoft TEAMS platform. Previous work reported the possibility of reaching moderate aerobic intensity while performing muscle-strengthening activities using weight machines [29] or body weight without specialized equipment [30]. The current study offers an online muscle-strengthening program using no equipment designed to address most cited barriers of adherence to regular participation in exercise, such as lack of time [29,30], costs [31], required transportation [31], fitness requirements [32], technical skills [33], and gender preferences [34]. Before starting the 12-week program, each participant received an in-person educational session explaining and demonstrating all the exercises. The first four weeks of the sessions were fully supervised. Participants were eased into the program using a three-week progressive start, completing 120 min of exercise in week one, 150 min in week two, and 180 min in weeks three and four. Participants were supervised by a certified trainer 3*X*/week for the first four weeks, then 2*X*/week for the next four weeks and 1*X*/week for the remaining four weeks. This strategy has successfully increased participant autonomy before they start the unsupervised phase [35]. At each session (See Fig. 1), a participant performed the four prescribed exercises as many times as possible (i.e., squats, tricep dips, lunges, and push-ups) for 45 s each, then switched immediately (15 s) to the next exercise followed by 1-min of rest at the end of each circuit. The circuit was repeated until the session was completed. Modifications were made if a participant had restrictions that prevented him from performing an exercise.

### 2.3 Data analysis

The transcripts were uploaded to the qualitative data analysis software package Nvivo QSR International where the interviews were coded using thematic analysis [36]. Thematic analysis was selected as the method for analysis because of its ability to tease out the experiences, thoughts, and behaviours across a data set [37]. After the first initial reading of each transcript, short summaries were drafted in the Nvivo 12 memos section, which contained brief descriptions of salient points, quotes of interest, and particularly noteworthy comments about the transcript. During the second reading of each transcript, line-by-line coding was used to support the formulation of common themes. Codes refer to a substantive piece of information or, as Boyatzis [40] summarized, “the most basic segment, or element, of the raw data or information that can be assessed in a meaningful way regarding the phenomenon.” Coding classifies the data to be compared systematically across a data set. LT was responsible for the data coding but was continuously in contact with MK. When disagreement occurred, DRB was deciding during weekly meetings. Although the questions were guided by the transtheoretical model [41], the grounded theory approach to report the findings. Thus, the results were not reported on the model but focused on the reported themes.

During the third reading of the interview transcripts, each interview was compared and contrasted with the initial 21 codes reported from within the study [38]. The codes were then confirmed, condensed, or modified into six themes. A theme is a “patterned response or meaning” [36] derived from the coded data. Using an inductive approach, where themes emerge from the coded data [39], the data were analyzed thematically to make comparisons within and across interviews [36]. After reviewing the six remaining themes, these were further condensed into two main themes, each containing two sub-themes. During the fourth and final reading of the transcripts, it was identified that all categories had been saturated and identified no additional themes.

## 3 Results

In seeking to understand the unique experiences of men living with obesity who participated in an online exercise program, the findings are organized into two main themes: those that removed barriers to exercise and those that improved the enablers of exercise. According to our participants, eliminating barriers meant not purchasing specialized equipment and not travelling to a gym facility. For our participants, the enablers to their success with the program included the structured format of the circuit program and having supervised sessions. These two primary themes are summarized below, with their associated sub-themes explained with direct quotes extracted from the participants' interview transcripts. Out of the 24 supervised sessions during the 12 weeks of the trial, participants attended an average of 22.8 ± 1.3 sessions or 95 %.

### 3.1 Removal of barriers

The first of the two themes to emerge from the data concerned the removal of barriers to participation. For the male participants in our study, removing barriers meant they did not need to purchase any equipment, and there was no participation cost. The removal of the travel barrier meant they could exercise in their homes and a space they considered safe.

### 3.2 No purchase necessary

All participants commented positively that they did not need to purchase any special exercise equipment or incur additional costs to participate in the program. The program was designed to be easy to perform and require only a mat and a chair with no additional purchases of specialized exercise equipment. There was also no cost to enrol in the program. Shawn, a 48-year-old data analyst, articulated what removing barriers meant to him.

So that's another barrier that is out of the way - it's not complicated. And it doesn't require any purchase of any equipment other than a mat, which I already had. And a pair of shorts and sneakers, which I already have, too. So, all of those barriers are out of the way, and I don't need to go anywhere. I don't need to purchase any equipment; I don't need to do anything complicated. All I need to do is commit the time and do the work. (Shawn).

Removing any additional costs to participation was an important factor for the compliance and attendance of the men in our study.

### 3.3 Easy access

All of the participants talked about the convenience of not having to travel to exercise. Accessing the online circuit program, similar to what would have been provided at a gym facility, was an important barrier for our participants to remove. Jake, a 42-year-old university professor, talked about the benefits of accessing an online program and felt he got a complete workout at home.

I liked the idea that during the pandemic, when we're all confined to home offices and not getting out as much, maybe there's a way to do short exercises at home that would get me the same kind of thing that I would otherwise get in the gym. (Jake).

Not having to travel meant the exercisers were able to work out in their own home and in a space that they considered safe. The program was designed to utilize a minimum amount of space, about 2 m [2] and only a chair or elevated surface as equipment. Having the physical space to complete the program, Dane, a 56-year-old retired military member, commented that he didn't need a gym facility to execute the program, leaving him very few excuses not to complete the sessions.

I thought it was a great program and something you can do in very little space. You don't need a gym. You don't need any weights … you need very little to do it; there are no excuses for not doing the exercises.

### 3.4 At home

The ability to do the program at home was a common thread among all the participants. Shawn talked about the convenience of having the program available in his home.

I can do it here in the house; I didn't have to go anywhere, or get the car warmed up and go out in the winter or go anywhere … all I had to do was dedicate the time and show up … it made it really easy to do the work. Because there's no, there are no real barriers to do that because I was already working at home. And I was already here … no joke, I went 15 feet into another room and there I was ready to go … it just made it very easy from just committing the time, and I had the space in the house to do it.

Seven of the ten participants were employed full-time and had young children living in their homes, suggesting that home life could sometimes be hectic. Thus, having the accessibility of an at-home circuit workout program was important to our participants, especially Rowan, a 36-year-old engineer.

I mean, personally, I'd jump to the basement and, you know, go to a quick exercise session, as I could do right after work, and you know, still have supper on the table for 5:30. If it was like, hey, come join this group, at the [local gym], and, you know, get in your gym clothes and be there at 10 o'clock or something. That might be a bit more difficult to actually undertake, rather than having it available in your house.

One of the interview questions asked if any of the men had ever participated in an online exercise program. All ten participants indicated they had never utilized an online exercise program before and that our study was advertised as exclusively online was an incentive to join. Shawn said that travelling to the gym would have been a barrier for him.

If I had to travel to the [local gym] if I had to travel to anywhere to be in person, that probably would have become a barrier. I think the online thing was actually a benefit regardless of the state of the world; the online supervision and doing this at home was an absolute thumbs up from me.

### 3.5 Safe space

Shawn also brought up an interesting point about being self-conscious and having to exercise in a gym and in front of or with others. Similar to Ref. [40] reported in their research on individuals living with obesity, Shawn was clear that having a safe space for him to exercise was critical in removing any excuse not to do the program.

Being able to do it in my house, away from others, I may have been self-conscious about doing this in front of people, but online; nobody's looking at you. So, there's no, you know, there's no real scrutiny or judgement from others, or, you know, you're basically on your own, in a safe place without any barriers, from equipment to having to travel or anything like that, it's basically you just need to go into the room and do it. There's nothing really more to it. So it takes away all the things that somebody could use as an excuse.

The male participants in our study had an 89.4% rate of compliance in attending all the sessions of their 12-week online circuit-style program. Each participant commented on the removal of barriers to exercise as a key contributor to their compliance and the overall success of the program. Shawn stated it well when he said;

It's relatively easy to do, other than the hour of actually doing the work. It's, you know, it's exercise. You get breathing hard and that, but there's no, there are no other barriers to it. It's just going in the room before supper and do it for an hour. Or you could just sit in the chair and waste that hour away.

### 3.6 Enhancing enablers

The second of the two primary themes pertained to enhancing the enabling factors to exercise compliance for our participants. For our male exercisers, having a well-structured program and one that was supervised was important to their success with the program.

### 3.7 Structured program

The online circuit program was structured around four prescribed exercises (i.e., squats, tricep dips, lunges, and push-ups) for 45 s each, then switched immediately (15 s) to the next exercise, followed by 1 min of rest at the end of each circuit. The circuit is repeated until the session is complete. For the men in our study, a structured program was one with a set start and end time and a simple program design. Dane said, *“this program gave me the structure I was looking for. Because doing it on my own it was, well, ‘I'll do it tomorrow’ kind of thing. Which never happened.”* Dane felt that having access to a structured program kept him more accountable than when he was left to work out on his own. He also liked the inclusive nature of the program; he continued by saying, *"it's all you put into it is what you're going to get out of it. But that's what's good about the program is that everybody can participate in the class."*

Maverick also agreed regarding the inclusivity of the program.

I always found that circuit training, you know, appeals to the mass because I could be working out with somebody who's much fitter than me, and both of us would get something out of the workout. So, I think that's what appealed to me.

### 3.8 Program design

The program was designed around functional strength exercises that were easy to execute in a small space with limited equipment. Beau, a 45-year-old engineer, had extensive past exercise experiences that indicated he was quite knowledgeable about creating his fitness routines but conceded that as he aged, his life became increasingly more complicated. He welcomed the opportunity to be supervised through a program he didn't create.

I'm comfortable that I could design my programs, but it, it's nice to kind of have it handed to you or set up and scheduled for you.

Sam, a 22-year-old full-time university student, also praised the design of the program and the benefits it gave him.

It was good because it wasn't extremely long … it was only an hour or less. It was perfect for a tight schedule. It was good because it was easy to exercise and easy to follow, with just 10–15 s of rest in between. I felt how my heart rate increased, and I was sweating. I was losing weight. It was amazing.

Aidan found both challenge and success with the program's repetitiveness and duration.

It [the program] had a certain amount of repetitiveness to it … I think everybody was aware that it would get potentially repetitive. I thought it was appropriate. And I thought it was it was well-balanced. And it was challenging enough … because the duration of it is what made it especially challenging.

### 3.9 Time

The participants had to schedule their workouts a week in advance to ensure a trainer would be available to them on the online platform. Matiss, a 33-year-old professor, stated that the trainers expecting him to attend his required sessions provided the right amount of motivation for him.

There was a scheduled time, and I promised somebody I would be there. And they were expecting me. And, like, I was usually the only one that chose the eight o'clock in the morning one. So, I was the only participant, and if I didn't show up, then [the trainer] would be just sitting there. So, I felt bad about that. I liked knowing that someone was expecting me.

Dane concurred that structured time was paramount for his continued attendance and completion of the program.

I found that the structure was better because it was at a specific time, and I just made time to do that. But what I find with, like right now, like the other night, seven o'clock came, I was busy. So, well, ‘I'll do it later’ and you know, eight-thirty came. Okay, well, that was too late to do it. So, I ended up doing it the next morning. But you start to slide that way easily. With a structured one, you know when your time was, and it is easier to maintain. (Dane).

### 3.10 Supervised sessions

At the beginning of the intervention, each participant was required to declare their supervised workout sessions for the upcoming week, thus ensuring they got their circuit times booked and a trainer would be available for the allotted time on the online platform. Maverick, a 58-year-old retired military member, stated that *“when this [online program] came up, I said, 'that's what I'm looking for. I'm looking for something structured,’ because I'm trying to do stuff on my own and it wasn't working for me.”* By structured, Maverick is referring to the scheduled times he had to commit to for his supervised exercise sessions.

For Dane, having the supervised portions of the program was the key to his success and his ability to create new habits.

For me, that's exactly what I was looking for and it was really good. What I did like about it was the supervised parts. Because they were supervised, I did them three times a week and then after a while, I went down two times a week and one time a week, I missed the supervised portions of it. And I still did it, like three times a week and everything and I still do, but I missed the supervised portions of it.

### 3.11 Expected attendance

The participants expressed feeling accountable for showing up to their scheduled sessions because the trainers expected them to be there. Beau, a 44-year-old engineer, concurred that the commitment to the supervised portions was critical to his 100% attendance record by saying, *“once I've committed to something, then I tend to stay at it. And it really helps that it's a group and that it is supervised.”* In fact, Aidan, a 38-year-old project manager, found it increasingly difficult to complete the workouts on his own after the supervised sessions had ended.

The two supervised sessions was still fine, but the one supervised session, I started slipping a bit, and since the no-supervised sessions hit, I've slipped quite a bit for most of the summer. Like I am doing none of the program now. (Aidan).

Rowen echoed similar sentiments that his compliance with the program dropped off after he was no longer accountable to anyone external.

Those first few weeks were very good because, you know, it was three times a week … and I found myself starting to slip towards the end of the project because I wasn't accountable to anyone; it was just kind of expected that I would continue doing it and I didn't. (Rowen).

### 3.12 Motivation

The participants talked about the trainers' adding motivation to their workouts by providing technique corrections and other encouraging cues. When asked what Jake thought of the trainers, he said, *“Oh, it's good; I like the direction. If I'm not doing a particular exercise correctly, he'll [the trainer] tell me to bend a little lower, push that right knee lower. It was motivational and helpful."*

Tristan, a 32-year-old social worker, also liked the attention and support offered by the online trainers.

I think because you had someone there counting down the timer, right? So that meant not having to do the extra paying attention to the time. Also, having someone there to talk to you through some exercises and be encouraging. So that was a bit of support to me.

Beau summed up his thoughts on what the trainers brought to the online format and that although each participant worked out in their own home, the trainers made it feel like a group atmosphere.

You still felt part of a group, and it was a group because there was a trainer there running your timings telling you when to start and stop, correcting you on things and not just correcting but boosting your morale along the way, too. And the feedback was, I found, really helpful.

## 4 Discussion

The results from our interviews suggest that by removing the barriers and enhancing the enablers to exercise, it is possible for men living with obesity to succeed with an online exercise program specifically designed for them. This study also suggests that exercise programs specifically targeting men would also interest men living with obesity. Our study identified that barriers to participation are cost and access to the program, whereas the enablers are the structured and supervised sessions of the program. Our results add to the current literature on men living with obesity, as not enough is known about the barriers and the enablers to success with online exercise programs for these men.

Our study identified novel ways to connect with men living with obesity who wanted to participate in an exercise program. Specifically, using an uncomplicated circuit-style program that required no equipment to purchase and very little space to execute the exercises was seen as desirable for these men. This format made it highly accessible to our participants. Jones and Nies (1996) also reported that increasing access to exercise increased compliance with an exercise program in their study of older African-American women [41].

Men living with obesity in our study preferred to exercise in non-judgemental spaces. The notion of stigma-free zones was vital to help prevent obese individuals from feeling “uncomfortable” and “self-conscious” when engaging in activity [12]. For our participants, non-judgemental spaces meant they preferred their home environment. This finding further suggests there could be opportunities for developing more online exercise programs for home use targeted specifically at men of higher weights. This is somewhat surprising as it is intuitive to think that men would not be bothered by a judgemental space [42].

Our study also identified enhancing the enablers to exercise was critical for engagement with an exercise program. It was consistent with Elliot et al. (2020) previous research on the experiences of men living with obesity accessing weight loss services. A key finding from Elliot et al. (2020) is that men often see weight loss services as feminized spaces; thus, tailoring the program specifically to men is critical in men's adoption of the exercise intervention. Our findings echo the need for male-targeted health promotion and exercise programs similar to Coles et al. (2010) reported in the UK [6] also indicated that increasing access to exercise increased compliance with the program in their study, where they reported a home-based training program reduced barriers to exercise in individuals living with diabetes ^47^.

Finally, our results also suggest that men living with obesity are motivated to continue with online exercise programs if they are externally accountable to others, specifically a motivating trainer who organizes the sessions. Strong support suggests that providing continuous sessions will further ensure greater and longer compliance with the exercise program.

## 5 Limitations

Although the current study provides insight into removing barriers and enhancing the enablers to increase adherence to regular exercise in men living with obesity, some limitations need to be acknowledged. First, the initial sample size was calculated based on the study's main outcome, and therefore, the sample size for the qualitative portion was affected. We must also acknowledge the limitations in generalizability to other populations, such as women living with obesity or individuals facing chronic conditions associated with obesity. It is also important to note that participants had to live in the surroundings of the laboratory for baseline testing, needed internet access at home, be technology literate, and the space to exercise. Considering the study conducted during the COVID-19 pandemic, we acknowledge the research's unique circumstances. There might have been differences in outcomes if the study was conducted outside of the pandemic period. The urgency and health concerns associated with the COVID-19 pandemic may have motivated participants differently than in non-pandemic times. Participants may have been more committed to the program offered online during the pandemic due to the uncertainty and isolation it brought. Also, the pandemic introduced significant stressors into daily life. Participants may have viewed the online exercise program as a coping strategy during this stressful period.

## 6 Conclusion

We explored the experiences of ten men living with obesity who participated in a 12-week online exercise circuit program. Our study suggests that removing barriers and enhancing the enablers increased compliance to the exercise program for men living with obesity. Given that there is limited evidence to support interventions that lead to long-term sustained change in health and behaviour regarding obesity, future research would benefit from an empirical examination of the results of continued supervised sessions over a longer time frame if that intervention leads to physiological benefits, and if the intervention would attract as much interest outside of the COVID-19 period, or for women living with obesity. This study is important because it offers facilitators to implement a specific online exercise program suited for males that leads to adherence to the current physical activity guidelines.

## Funding

The 10.13039/100004411Heart and Stroke Foundation of New Brunswick funded this project.

## Declaration of competing interest

The authors declare that they have no known competing financial interests or personal relationships that could have appeared to influence the work reported in this paper.

## Data Availability

Data will be made available on request.
