# Supplementary material for: Online exercise program for men living with obesity: Experiences, barriers, and enablers
Source: Contemp Clin Trials Commun. 2023 Nov 10;36:101226. doi: 10.1016/j.conctc.2023.101226 (PMC10681938; doi:10.1016/j.conctc.2023.101226)
Supplement: Multimedia component 1 [file mmc1.docx]

INTERVIEW

GUIDE

Thank you for participating today. This interview aims to investigate your experience with the online exercise program. As a participant in this study, please take your time; don’t feel rushed. If you are struggling to find the words to describe the situation, take a moment to think about it. You can ask to stop the interview at any time. There is no right/wrong answer; I am trying to gain insight into your experiences, and you are the expert. The interview will be audio recorded, so please speak clearly. Let’s begin.

**Before the program**

1. Can you tell me why you were interested in participating in this study?
2. How motivated were you to start the circuit program?
   1. What helped motivate you? What prevented you from being motivated?
3. Have you tried resistance/circuit training/to be more active in the past?
   1. Was there something different from your past experiences aiming to be more active?
   2. Is there something different this time?
4. How important was it for you that this program targeted higher-weight males specifically?
   1. Can you tell me more about that? How is this the same/different from prior exercise programs you’ve participated in?
   2. How important was the gender aspect for you? How important was the weight aspect?
5. What are your thoughts about the online aspect of the program?
6. How did you feel about the program being guided?

**Exercise Program**

1. What motivated/encouraged you to attend sessions in the first 12 weeks when you received supervision?
2. What was the circuit program like for you?
   1. Easy or hard for you? What made it easier or harder? How has that changed over time?
3. Have you gained anything from participating in this program?
4. In what ways did this program meet your expectations? In what ways was it different from what you expected?
5. What would you want to change about this program?
6. Were you concerned that you might not be able to complete the program physically?
7. How safe did you feel doing the circuit program?
8. Do you feel this program is suited for higher-weight males?

How did you feel about the online aspect of the program?

**After 24 weeks**

1. Do you feel you improved any health elements?
   1. If so, which ones? If not, why not?
2. Are you still doing some resistance circuit sessions on your own?
   1. Why (why not), how often?
3. How confident can you maintain your current Physical Activity (PA)level in the next 6 months?
   1. What would help you maintain your current PA level? What might get in the way?
4. What kinds of exercise programs would you be interested in in the future (if any)?
   1. How appealing is another
      1. Higher-weight, male-only exercise program?
      2. Circuit-training program?
      3. Guided-exercise program?
      4. Online exercise program?
      5. In-person, guided exercise program?
   2. Can you tell me more about feeling that way?
5. Anything else you want to share about your experience performing the circuit training?

Thank you for participating!
